# Genotyping and Subtyping *Cryptosporidium* To Identify Risk Factors and Transmission Patterns — Nebraska, 2015–2017

**DOI:** 10.15585/mmwr.mm6912a4

**Published:** 2020-03-27

**Authors:** Brianna K. Loeck, Caitlin Pedati, Peter C. Iwen, Emily McCutchen, Dawn M. Roellig, Michele C. Hlavsa, Kathleen Fullerton, Thomas Safranek, Anna V. Carlson

**Affiliations:** ^1^Division of Public Health, Nebraska Department of Health and Human Services; ^2^University of Nebraska–Lincoln, Nebraska; ^3^Nebraska Public Health Laboratory; ^4^University of Nebraska Medical Center, Omaha; ^5^Division of Foodborne, Waterborne, and Environmental Diseases, National Center for Emerging and Zoonotic and Infectious Diseases, CDC.

*Cryptosporidium* is an enteric pathogen that is transmitted through animal-to-person or person-to-person contact or through ingestion of contaminated water or food. In the United States, *Cryptosporidium* affects an estimated 750,000 persons each year; however, only approximately 11,000 cases are reported nationally (*1*,*2*). Persons infected with *Cryptosporidium* typically develop symptoms within 2 to 10 days after exposure. Common symptoms include watery diarrhea, abdominal cramps, nausea, vomiting, or fever, which can last 1 to 2 weeks. Cryptosporidiosis is a nationally notifiable disease in the United States. Nebraska presents a unique setting for the evaluation of this pathogen because, compared with other states, Nebraska has a greater reliance on agriculture and a higher proportion of the population residing and working in rural communities. *Cryptosporidium* species and subtypes are generally indistinguishable using conventional diagnostic methods. Using molecular characterization, Nebraska evaluated the genetic diversity of *Cryptosporidium* and found a dichotomy in the distribution of cases of cryptosporidiosis caused by *Cryptosporidium parvum* and *Cryptosporidium hominis* among rural and urban settings. Characterizing clusters of *C. hominis* cases revealed that several child care facilities were affected by the same subtype, suggesting community-wide transmission and indicating a need for effective exclusion policies. Several cases of cryptosporidiosis caused by non–*C.* *parvum* or non–*C.* *hominis* species and genotypes indicated unique animal exposures that were previously unidentified. This study enhanced epidemiologic data by validating known *Cryptosporidium* sources, confirming outbreaks, and, through repeat interviews, providing additional information to inform cryptosporidiosis prevention and control efforts.

During September 2015–December 2017, a total of 630 *Cryptosporidium*-positive stool specimens were reported to public health agencies by clinical laboratories, which most commonly used culture independent diagnostic testing (CIDT) and enzyme immunoassays (EIAs) for detection; among these 630 positive stool specimens, 149 (24%) were sent to the Nebraska Public Health Laboratory (NPHL), and subsequently to CDC, where genotyping was conducted using nested polymerase chain reaction–restriction fragment length polymorphism analysis and DNA sequencing of the 18S rRNA gene and the gp60 gene (*3*,*4*). Epidemiologic data on cases with genotyped and subtyped *Cryptosporidium* stool specimens were exported from the Nebraska Electronic Disease Surveillance System and linked to molecular data to assess association among species and exposures. Odds ratios (ORs), p-values, and 95% confidence intervals (CIs) were calculated using SAS statistical software (version 9.4; SAS Institute). ArcGIS was used to map cases to depict geographic distribution of cases.

Among 149 patients with a molecularly characterized stool specimen, the median age was 22 years (range = 7 months–79 years); 79 (53%) patients were female. Eight patients (5%) were hospitalized, and no deaths were reported. Species and genotypes were identified in 149 submitted specimens, 80 (54%) of which were positive for *C. hominis* and 58 (29%) for *C. parvum*. Other identified species and genotypes included *Cryptosporidium chipmunk genotype I* and *Cryptosporidium felis* (three each); *Cryptosporidium ubiquitum* (two); and *Cryptosporidium canis*, *Cryptosporidium melargridis*, and *Cryptosporidium skunk genotype* (one each).

The 149 patients reported various exposures; 81 (54%) reported animal exposures, including 32 (40%) who reported exposure to dogs, 26 (32%) who reported exposure to cats, and 23 (28%) who reported exposure to cattle (Figure 1). Follow-up interviews identified specific dog, squirrel, and skunk exposures that previously had not been not mentioned. Overall, 11 *C. hominis* cases were associated with outbreaks. Three outbreaks were identified with the same *C. hominis* subtype within multiple child care facilities: 1) the first involved two cases, one each in two different facilities located in two distant local health department jurisdictions; 2) the second involved three cases, one case and two cases, respectively, in two child care facilities located in two neighboring local health department jurisdictions; and 3) the third involved six cases at one child care facility that is located within two adjoining local health department jurisdictions. *C. parvum* was associated with two outbreaks; however, only one stool specimen was sent to CDC.

**FIGURE 1:**
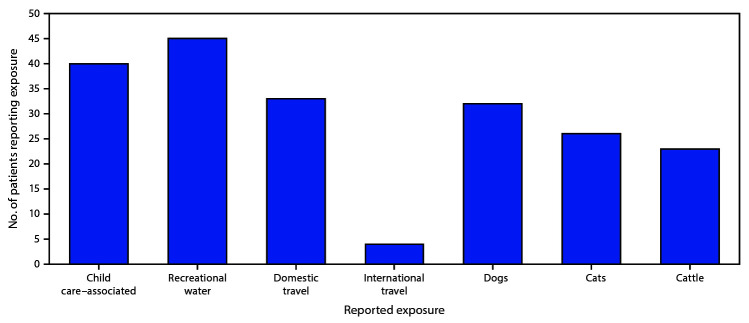
Exposures*^,†^ commonly reported by cryptosporidiosis patients (N = 149) — Nebraska, September 2015–December 2017 * Patients could report multiple exposures. No patient reporting dog exposure reported cattle exposure. ^†^ Does not include data from follow-up interviews that identified specific dog, squirrel, and skunk exposures not previously mentioned.

Patients with *C. parvum* infection were more likely to report exposure to dogs and cattle than were those infected with other species. Patients with *C. hominis* infection were approximately nine times more likely to have reported a day care or child care exposure than were patients infected with other species (Table). Potential associations among species and recreational water exposure were also examined, but not found to be significant.

**TABLE:** Associations among exposures and risk of infection with *Cryptosporidium parvum* and *Cryptosporidium hominis* species (N = 149) — Nebraska, 2015–2017

Species	Exposure	OR (95% CI)*
*C. parvum*	Dogs	3.88 (1.46–10.26)
	Cattle	16.04 (4.50–57.28)
	Recreational water	0.48 (0.21–1.12)
*C. hominis*	Day care or child care	9.55 (3.38–26.98)
	Recreational water	1.48 (0.68–3.20)

**Abbreviations:** CI = confidence interval; OR = odds ratio.

* Compared with non–*C. parvum* and non–*C. hominis* species.

Reported cases were mapped by county of patient residence to document the urban and rural distribution of *C. hominis* cases and *C. parvum* cases in the state. *C. hominis* cases were identified among patients from more highly populated urban counties (nearly half of *C. hominis* cases were reported from Douglas County, the most populous county), whereas *C. parvum* cases were identified among cases from more sparsely populated rural counties (Figure 2).

**FIGURE 2:**
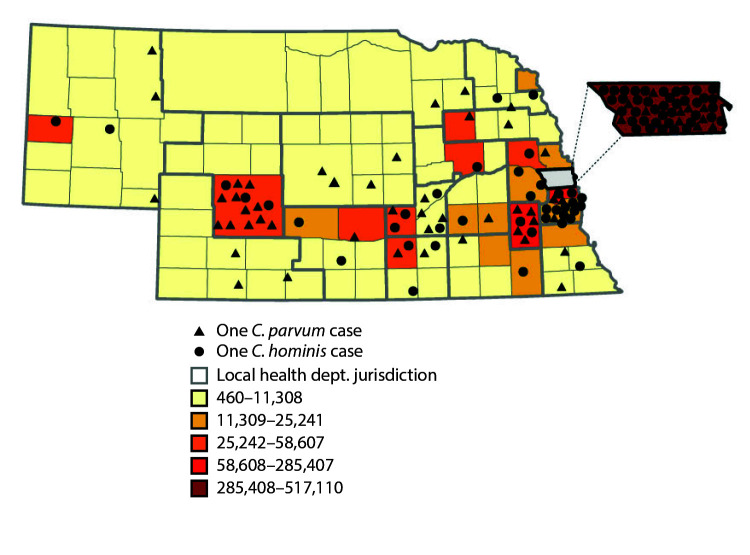
Distribution of *Cryptosporidium parvum* and *Cryptosporidium hominis* cases — Nebraska, September 2015–December 2017* * Placement of symbols within a county is random and does not indicate exact location of cases.

## Discussion

In 2010, CDC launched CryptoNet,* the first such surveillance system in the United States, with the objective of collecting and analyze molecular characterization and epidemiologic data for each nationally notified case of cryptosporidiosis (*5*). Collecting epidemiologic data such as exposures and risk factors and linking those data with molecular data on *Cryptosporidium* from clinical specimens can elucidate the transmission routes of *Cryptosporidium* in the United States. Nebraska has participated in CryptoNet since 2015 and uses molecular characterization data to enhance epidemiologic case investigations and inform prevention and control efforts. Whereas approximately 40 distinct C*ryptosporidium* species and genotypes are known, only approximately 20 have been reported to infect humans; species and genotypes are generally indistinguishable using conventional diagnostic methods, such as an ova and parasite examination (*6*). Testing and analysis in Nebraska identified eight species and genotypes. *C. hominis* and *C. parvum* are known to be the two species of *Cryptosporidium* that most frequently cause community outbreaks (*7*), and Nebraska data were consistent with this pattern and confirmed several known risk factors and distribution patterns by supplementing epidemiologic case investigations with genotyping and subtyping data.

The analysis indicated *C. parvum* cryptosporidiosis cases were associated with animal exposures and occurred more frequently among persons who live in rural settings, whereas *C. hominis* cryptosporidiosis cases were more likely to be reported in residents of urban, populated areas. Contact with cattle and dogs were each significantly associated with *C. parvum* cryptosporidiosis cases. Previous studies of *Cryptosporidium* in dogs have demonstrated varying carriage rates (*8*); species commonly identified include *C. parvum* and *C. canis*. These data can be used to reinforce cryptosporidiosis prevention messages, including hand hygiene following contact with animals or their feces.

This analysis highlighted the association between child care facility exposure and *C. hominis* cryptosporidiosis cases, and clusters of *C. hominis* cases were identified with the same subtype among several child care facilities. This might indicate an unidentified common exposure outside of child care (e.g., swimming pool or waterpark) or the attendance of a child who was excluded from one facility because of gastrointestinal illness at a different child care facility, leading to the introduction of the pathogen in another facility and further community transmission. Such data can be used by local health departments and environmental health partners to inform exclusion policies and educate child care facilities about the person-to-person transmission of these pathogens and to assist facilities with implementing careful screening and assessments of symptomatic children.

Genotype characterizations also serve to inform public health investigations of potential risk factors and unusual exposures. For example, Nebraska’s unique cases of non–*C. parvum* or non–*C. hominis* species and genotypes provided the impetus for public health personnel to conduct follow-up interviews, which were able to identify previously unreported dog, squirrel, and skunk exposures. These data will be useful to identify potential geographic regions or populations that are more commonly affected by these less frequently reported species and genotypes.

The findings in this report are subject to at least three limitations. First, cryptosporidiosis might be undiagnosed or underreported. Second, the analysis included approximately one quarter of cases with *Cryptosporidium*-positive specimens that were sent to NPHL. However, this number likely is not representative of all occurrent cases, given that approximately 600 cryptosporidiosis cases were reported to the state public health department during September 2015–December 2017. Finally, stool samples from animals were not tested for *Cryptosporidium,* which could have linked clinical samples to animal samples and ultimately confirmed the source of transmission. The dog-contact risk factor could be confounded by dogs having rural exposure to cattle; however, this cannot be confirmed given that further follow-up on dog exposure was not investigated.

Public health surveillance for cryptosporidiosis is important to increase knowledge about risk factors and transmission patterns and to promote community cryptosporidiosis prevention education. The findings in this report provide insight into the patterns of human *Cryptosporidium* transmission in Nebraska and highlight the importance of collaboration between epidemiologists and laboratorians for improving and protecting the public’s health. Nebraska is continuing to explore ways to improve CryptoNet activities, such as further increasing sample submission to NPHL, increasing timeliness of interviews, conducting sequencing methods in real time rather than retrospectively, and reporting results to public health epidemiologists sooner.

**Summary**

**What is already known about this topic?**

Fecal-oral transmission of *Cryptosporidium* can occur following contact with an infected animal or person or through ingestion of contaminated water or food.

**What is added by this report?**

Molecular typing of *Cryptosporidium* in Nebraska during 2015–2017 found that *C. parvum* cases were associated with animal exposures in rural settings, whereas *C. hominis* cases were more likely to occur in urban areas. Several child care facilities affected by the same *C. hominis* subtype suggested community-wide transmission and indicated a need for effective exclusion policies.

**What are the implications for public health practice?**

Characterizing *Cryptosporidium* species, genotypes, and subtypes from urban and rural populations can improve outbreak detection and investigation, identify potential sources, and inform prevention strategies.

## Footnotes

* https://www.cdc.gov/parasites/crypto/cryptonet.html.
